# The role of costal cartilage in musculoskeletal regeneration: A systematic review

**DOI:** 10.1002/jeo2.70753

**Published:** 2026-06-16

**Authors:** Francesca Veronesi, Luca Cavazza, Leonardo Vivarelli, Marta Pluchino, Gianluca Giavaresi, Dante Dallari, Marco Govoni

**Affiliations:** ^1^ Surgical Sciences and Technologies IRCCS Istituto Ortopedico Rizzoli Bologna Italy; ^2^ Reconstructive Orthopaedic Surgery and Innovative Techniques‐Musculoskeletal Tissue Bank IRCCS Istituto Ortopedico Rizzoli Bologna Italy

**Keywords:** bone regeneration, cartilage regeneration, clinical studies, costal cartilage, in vivo studies, osteochondral defect regeneration

## Abstract

**Purpose:**

Musculoskeletal tissue regeneration remains a major clinical challenge due to the limited intrinsic healing capacity of cartilage, bone and interface tissues, as well as the complexity of recreating their hierarchical structure and mechanical properties. In recent years, costal cartilage (CC) has emerged as a promising and versatile resource for regenerative applications, owing to its hyaline cartilage composition, biological plasticity and relative surgical accessibility. This systematic review aims to critically evaluate in vivo and clinical evidence published over the last decade regarding the use of CC‐derived cells and matrices for musculoskeletal tissue regeneration.

**Methods:**

A systematic search of PubMed, Scopus and Web of Science databases was conducted following Preferred Reporting Items for Systematic Reviews and Meta‐Analyses (PRISMA) guidelines, identifying 26 eligible studies, including 18 in vivo and 8 clinical investigations.

**Results:**

CC was employed either as a source of costal chondrocytes or cartilage‐derived stem cells, or as decellularized or particulate extracellular matrix scaffolds. Preclinical studies demonstrated consistent regenerative potential across cartilage, osteochondral, bone and enthesis models, with outcomes showing hyaline‐like tissue formation, improved biomechanical properties, enhanced integration with host tissue and coordinated chondrogenic and osteogenic responses. Clinical studies, primarily focused on cartilage and osteochondral defects, reported significant improvements in patient‐reported outcomes, imaging‐based repair quality and functional recovery, with follow‐up periods extending up to 5 years and a favourable safety profile.

**Conclusions:**

Cell‐based CC approaches showed robust biological efficacy but were associated with greater regulatory and translational complexity due to substantial manipulation requirements. In contrast, matrix‐based CC strategies offered a more readily translatable option, leveraging the intrinsic bioactivity of the extracellular matrix while reducing regulatory burden. CC could be considered a highly promising and adaptable platform for musculoskeletal regeneration. However, heterogeneity in study design, limited sample sizes and variable methodological quality highlight the need for standardized protocols and well‐designed, long‐term randomized clinical trials to definitively establish clinical indications and optimize therapeutic strategies.

**Level of Evidence:**

N/A.

**Abbreviations**
ACAN: Aggrecan gene
ADL: activities of daily living
AOFAS: American Orthopedic Foot and Ankle Society score
ARMAB: Advanced Regenerative Medicine and Advanced Biological Products
Asc‐2P: ascorbate
ATMPs: Advanced Therapy Medicinal Products
AUC: auricular cartilage
BMSCs: bone marrow‐derived mesenchymal stem cells
BV: bone volume
CC: costal cartilage
CChons: costal chondrocytes
CDSCs: costal‐cartilage‐derived stem cells
COL1A1: collagen Type I alpha 1 chain gene
COL2A1: collagen Type II alpha 1 chain gene
COLL I: Collagen I
COLL II: Collagen II
DTS: decellularized tendon scaffold
ECM: extracellular matrix
EQ VAS: EuroQol visual analogue scale
FAAM: Foot and Ankle Ability Measure score
GAG: glycosaminoglycans
GelMA: gelatin methacrylate
HE: Hospital Exemption
HHS: Harris Hip Score
HTO: high tibial osteotomy
hWJMSC: human Wharton's jelly mesenchymal stem
ICRS: International Cartilage Repair Society
ICRS‐CRA: International Cartilage Repair Society‐Cartilage Repair Assessment
IKDC: International Knee Documentation Committee
KOOS: Knee injury and Osteoarthritis Outcome score
LCCM: lyophilized human CC matrix
MAD: mineral apposition distance
MA‐ECM: adipose‐derived ECM
MF: microfacture
MOCART: magnetic resonance observation of cartilage repair tissue
NSC: nasal cartilage
OCN: osteocalcin
PCHCA: particuled costal hyaline cartilage allograft
PCL: polycaprolactone
PICOS: Population, Intervention, Comparison, Outcomes, Study design
PR1P: prominin‐1‐derived peptide
PRISMA: Preferred Reporting Items for Systematic Reviews and Meta‐Analyses
QOL: quality of life
RCT: randomized controlled trial
RoB 2: Risk of Bias 2.0
RUNX2: runt‐related transcription factor 2 gene
SD: subcondral drilling
SDSCs: synovial‐derived stem cells
SF: silk fibrin
SOX9: SRY‐box transcription factor 9 gene
UCLA: University of California Los Angeles scale

## INTRODUCTION

The regeneration of musculoskeletal tissues remains a major challenge in regenerative medicine, despite advances in cell biology and biomaterials. Bone, cartilage, tendon and skeletal muscle exhibit limited intrinsic repair capacity due to their complex hierarchical structure, low vascularity and a hostile post‐injury microenvironment characterized by inflammation and fibrosis [2, 29, 50]. Clinical translation of cell‐ and tissue‐based therapies is further constrained by poor cell survival and engraftment, age‐ and passage‐related loss of cellular potency and the difficulty of recreating the biochemical and biomechanical gradients of native tissues [17, 48]. These challenges are particularly pronounced in cartilage and osteochondral defects and at musculoskeletal interfaces, where insufficient integration often leads to mechanically inferior repair tissue [14, 25, 52]. Consequently, although tissue engineering strategies show promise, the identification of optimal cell sources, scaffold architectures and biological cues remains a critical barrier to clinical translation.

In this context, costal cartilage (CC), the most abundant permanent cartilaginous tissue in the human body, has gained increasing attention as a potential resource for musculoskeletal tissue regeneration. CC represents a reservoir of hyaline cartilage with biochemical composition and structural characteristics comparable to those of articular cartilage and growth plate cartilage [11, 23]. Its aneural and avascular nature, together with its relative surgical accessibility, results in reduced donor‐site morbidity compared with harvesting bone or articular cartilage [40]. Moreover, costal chondrocytes (CChons) and costal‐cartilage‐derived stem cells (CDSCs) exhibit high proliferative capacity, phenotypic stability and multilineage differentiation potential toward chondrogenic, osteogenic and tenogenic lineages, making them attractive candidates for bone, cartilage and bone–tendon interface regeneration [3, 56]. In vitro studies further demonstrate that CChons yield higher cell numbers than articular chondrocytes, while maintaining the ability to synthesize hyaline‐like matrix components and superficial zone proteins [16].

Nevertheless, cell‐based approaches relying on isolated and expanded chondrocytes or stem cells typically involve manufacturing process classified as substantial manipulation. As a result, these therapies often fall under stringent regulatory frameworks, such as Advanced Therapy Medicinal Products (ATMPs) or, in specific national contexts, non‐commercial pathways including Hospital Exemption (HE), substantially increasing development complexity, costs and regulatory burden [10, 42].

Conversely, CC can also be exploited as a natural scaffold, particularly in its decellularized form. Owing to the intrinsic mechanical properties and bioactive composition of its extracellular matrix (ECM), decellularized CC supports chondrogenesis and tissue regeneration without the need for exogenous cells or growth factors. Although CC undergoes age‐related hypertrophy and progressive ossification, this characteristic may be advantageous in regenerative settings by conferring responsiveness to induced ossification processes, especially in osteochondral or bone repair applications [41].

From a regulatory standpoint, allogeneic decellularized CC matrices or scaffolds are generally classified as tissues or allografts (Human Cell, Tissue, and Cellular and Tissue‐Based Products, HCT/Ps) under tissue banking regulations. This classification enables broad availability from cadaveric donors while eliminating donor‐site morbidity associated with autologous tissue harvesting [35].

Importantly, CC‐derived tissues and products are already well established in clinical practice for craniofacial reconstruction, otoplasty and tracheoplasty [1, 19, 32]. Building on this established clinical track record, recent studies increasingly propose the use of CC, both in its cellular and decellularized forms, as a promising platform for the development of novel tissue engineering strategies aimed at the repair of bone, chondral and osteochondral defects. Accordingly, the aim of the present systematic review is to collect and critically analyse literature published over the last decade on the use of CC, in the form of both CC‐derived cells (CChons and CDSCs) and CC‐derived matrices, for musculoskeletal tissue regeneration.

## MATERIALS AND METHODS

### Search strategy

To design this systematic review, the PICOS (Population, Intervention, Comparison, Outcomes, Study design) model was used. More precisely, the following studies were considered: those involving surgically induced animal models or patients with traumatic and/or degenerative cartilage and bone disorders, minimal bone loss or minimal tendon damage, who underwent the implantation of CC‐cells, CC‐matrices or scaffolds (intervention) and were compared with untreated patients or with patients before and after implantation surgery (comparison). In vivo and clinical trials of all types (study design) were considered. In addition, the included studies ranged from December 2015 to December 2025 and were written in the English language.

This review included a systematic search of three databases (PubMed®, Scopus and Web of Science™), which began in October 2025 and was updated monthly until December 2025. This was carried out in accordance with the Preferred Reporting Items for Systematic Reviews and Meta‐Analyses (PRISMA) statement [31].

The term used were the followed: ((‘Costal Cartilage’ [Mesh] OR ‘costal cartilage’ OR ‘rib cartilage’ OR ‘costochondral cartilage’) AND (‘Tissue Engineering’ [Mesh] OR ‘Regeneration’ [Mesh] OR ‘tissue regeneration’) AND (‘Musculoskeletal System’ [Mesh])) (Table 1).

**Table 1:** Combination of free vocabulary and/or Medical Subject Headings (MeSH) terms for the identification of studies.

*PubMed*: ((‘Costal Cartilage’[MeSH Terms] OR ‘Costal Cartilage’[All Fields] OR ‘rib cartilage’[All Fields] OR ‘costochondral cartilage’[All Fields]) AND (‘Tissue Engineering’[MeSH Terms] OR ‘Regeneration’[MeSH Terms] OR ‘tissue regeneration’[All Fields]) AND ‘Musculoskeletal System’[MeSH Terms]) AND ((2015/10/28:2025/10/28[pdat]) AND (english[Filter]))
*Scopus*: (TITLE‐ABS‐KEY (costal cartilage OR rib cartilage OR costochondral cartilage) AND TITLE‐ABS‐KEY (tissue engineering OR regeneration OR tissue regeneration) AND TITLE‐ABS‐KEY (musculoskeletal system)) AND PUBYEAR > 2015 AND PUBYEAR < 2025 AND (LIMIT‐TO (LANGUAGE, ‘English’)
*Web of Science Core Collection:* (TITLE‐ABS‐KEY (costal cartilage OR rib cartilage OR costochondral cartilage) AND TITLE‐ABS‐KEY (tissue engineering OR regeneration OR tissue regeneration) AND TITLE‐ABS‐KEY (musculoskeletal system)) and 2025 or 2024 or 2023 or 2022 or 2021 or 2020 or 2019 or 2018 or 2017 or 2016 or 2015 (Publication Years) and English (Languages)

The in vitro studies, studies using CC for the regeneration of non‐musculoskeletal tissues, studies using chondrocytes collected from a site other than the rib, reviews, letters, comments to editor, meta‐analysis, editorials, protocols and recommendations and guidelines were excluded.

The study selection was performed after submitting the articles to EndNote to eliminate duplicates. The relevant articles were screened by reading the title and abstract by two authors (F. V. and L. C.). Studies that did not fulfil the inclusion criteria were excluded from the review. Any discrepancies were resolved through discussion until consensus was reached, or, when necessary, with the involvement of a third reviewer (M. G.). The remaining studies were then included in the final data extraction phase, and the reference lists of the selected articles were also examined.

Relevant data were independently extracted and collected using a standardized extraction form by two authors (F. V. and L. C.), and the information was included in Tables 2 and 3. They include references (Ref.), animal species and number and type of defect, type of CC matrices or cells, experimental groups, follow‐up (F‐up) and materials and methods, and main outcomes (Table 2); References (Ref.), type of study, number of patients, and type of lesion, type of CC matrices or cells, experimental groups, follow‐up (F‐up) and materials and methods and main outcomes (Table 3).

**Table 2:** Characteristics of the in vivo studies included in the systematic review.

Ref.	Animals defects	CC matrices/cells	Experimental groups	F‐up: Mat and met	Main outcomes
*Orthotopic models*					
Ryu 2022 [33]	12 beagle dogs. Full‐thickness defect (6 × 2 mm) in MFC	CC‐matrix: Human lyophilized CC powder	1: No treatment (three dogs) 2: CC‐Matrix (three dogs) 3: MA‐ECM (three dogs) 4: CC‐matrix/MA‐ECM (three dogs)	8 mo: ‐ MRI ‐ Compressive strength ‐ Histology ‐ IHC ‐ ELISA	**Groups 3 and 4**: ↓ MMP3, MMP9; ↑ COLL II than **Groups 1 and 2** **Group 4**: ↑ macroscopic cartilage‐like appearance, average stiffness, average stress at the relaxation, volume fill, ICRS score than **Groups 1–3** **Group 4:** ↑ MOCART score; ↓ COLL I than **group 1**
Changchen 2021 [7]	12 rabbits. 4th–7th CC defect on the right side	CC‐matrix: Autologous CC	1: No treatment (four rabbits) 2: CC‐matrix (four rabbits) 3: Autologous decellularized AUC (four rabbits)	4 mo: ‐ Thoracic CT ‐ Histology ‐ RT‐PCR	**Groups 2 and 3**: ↑ newly‐formed tissue, Sox9, Acan, Col2α1, Runx2; ↓ Col1α1 than **group 1**
Han 2021 [18]	Six NZW rabbits. fifth CC defects (1.5 cm lengths) on the right and left sides	CC‐matrix: Autologous CC	1: No treatment on the right (six rabbits) 2: CC‐matrix on the left (six rabbits)	4 mo: ‐ Macroscopic evaluations ‐ Biomechanical evaluations	**Group 1**: Macroscopic appearance, stress‐strain relationships as **Group 2**
Zheng 2024 [55]	18 SD rats. Femoral groove OC defect (1.5 × 1 mm)	CC‐matrix: ECM synthesized by autologous CChons	1: No treatment (six rats) 2: CC‐matrix (six rats) 3: Scaffold + Asc‐2P (six rats)	3 mo: ‐ Nanoindentation tests ‐ Histology ‐ IHC	**Groups 2 and 3**: ↓ hardness, stiffness, COLL I, COLL X; ↑ Elastic modulus, COLL II, GAG, Aggrecan than **Group 1** **Group 3**: Gross appearance; ↑ Hardness, stiffness, elastic modulus, COLL II than **Group 2**
Lee 2023 [26]	24 NZW rabbits. Femoral groove OC defect (5 × 2 mm)	CC‐matrix: Human decellularized CC mixed with a crosslinked HA‐CMC carrier	1: No treatment (six rabbits) 2: MF (six rabbits) 3: MF + CC‐matrix (six rabbits) 4: Sham (six rabbits)	6 mo: ‐ Gait analysis ‐ MRI ‐ Gross morphology ‐ Histology ‐ IHC ‐ Biomechanical tests	**Group 3**: Stance and swing phases; ↑ complete and uniform defect filling, GAG and collagen deposition, COLL II, stiffness, hardness, elastic moduli; ↓ COLL I than **Groups 1 and 2**
Du 2015 [13]	21 Japanese white rabbits. Femoral groove OC defect (5 × 3 mm)	CC‐matrix: Autologous CC	1: No treatment (14 knees) 2: Flat‐bottom CC‐matrix (14 knees) 3: Mid‐cut CC‐matrix (14 knees)	3 mo: ‐ Macroscopic evaluation ‐ Histology ‐ IHC	**Groups 2, 3**: ↑ Macroscopic and histological appearance, continuous integration between scaffold and host bone, COLL II than **Group 1**
Higeuchi 2023 [21]	36 Wistar rats. Mandibular angle defect (4 mm diameter)	CC‐matrix: Autologous CC	1: No treatment (nine rats); 2: CC‐matrix (nine rats) 3: Autologous mandibular bone graft (nine rats) 4: Carbonate apatite graft (nine rats)	3 mo: ‐ CT ‐ Histology	**Groups 2 and 3**: laminar plate bone repair of the defect; ↑ BV than **Groups 1 and 4**
Zhao 2015 [53]	54 Japanese white rabbits. Full‐thickness cartilage defect (4 × 3 mm) in groove centre	CC‐cells: Autologous CChons (1 × 10^7^ cells/mL)	1: No treatment (18 rabbits) 2: Chitosan hydrogel (18 rabbits) 3: Chitosan hydrogel + cells (18 rabbits)	3 mo: ‐ Gross morphology ‐ Histology ‐ IHC	**Group 3**: ↑ macroscopic and histological cartilage regeneration, COLL II than **Groups 1 and 2**
Cao 2021 [5]	40 SD rats. Full‐thickness cartilage defect (2 × 3 mm) in groove centre	CC‐cells: Allogenic BMSCs + allogenic CChons (3:1)	1: No treatment (eight rats) 2: PCL + cells (eight rats) 3: PCL + TGFβ3 + cells (eight rats) 4: PCL/GelMA + cells (eight rats) 5: PCL/GelMA + TGF‐β3 + cells (eight rats)	3 mo: ‐ Macroscopic evaluation ‐ X‐ray ‐ Histology ‐ IHC ‐ Mechanical tests ‐ Gait analysis ‐ Gene expression	**Group 5**: ↑ macroscopic regeneration, COLL II; ↓ mechanical pain than **Groups 1–4** **Group 5**: ↑ Acan than **Group 1** **Groups 1 and 2**: unfilled defects **Group 2**: ↑ Young's modulus than **Groups 1 and 3–5** **Groups 3 and 4**: larger areas of neocartilage **Groups 3 and 5**: ↓ COLL X **Group 3**: ↑ Young's modulus than **Groups 1 and 5** **Group 4**: ↑ Acan than **Groups 1 and 2** **Groups 4 and 5**: ↓ COLL I than **Groups 1–3** **Groups 4 and 5**: ↑ Col2A1 than **Group 1**
Ma 2022 [27]	Nine SD rats. Femoral groove OC defect (1 × 1 mm)	CC‐cells: Allogenic CChons pellets (2.5 × 10^5^); CChons + SDSCs (75%:25%)	1: No treatment (three rats) 2: Cells (three rats) 3: Co‐culture (three rats)	1 mo: ‐ Gross evaluation ‐ Histology ‐ IHC	**Groups 2 and 3**: ↑ defect restoration, pellet integration and macroscopic and histological appearance than **Group 1** **Group 3**: ↑ COLL II; ↓ COLL X than **Group 2**
Zheng 2022 [54]	24 SD rats. Femoral groove OC defect (1.5 mm diameter)	CC‐cells: Allogenic CChons pellets (5 × 10^5^); CChons + WJMSC (2.5 × 10^5^:2.5 × 10^5^)	1: No treatment (six rats) 2: Cells (six rats) 3: hWJMSC (5 × 10^5^) (six rats) 4: Co‐culture (six rats)	3 mo: ‐ Gross evaluations ‐ Histology ‐ IHC	**Groups 2 and 4**: ↑ Macroscopic and histological appearance, COLL II than **Groups 1 and 3** **Groups 3 and 4**: ↓ COLL X than **Groups 1 and 2**
Cai 2025 [4]	18 C57BL/6 mice. Femoral segmental bone defect (4 mm)	CC‐cells: Allogenic CDSCs (1 × 10^7^ cells/mL)	1: Collagen hydrogel (six mice) 2: Collagen hydrogel + cells (six mice) 3: Collagen hydrogel + cells + PR1P (six mice)	2 mo: ‐ Micro‐CT ‐ Histology ‐ IHC ‐ IP ‐ TRAP staining	**Groups 2 and 3**: ↑ bone mass, cross‐sectional area of mid‐femur, OCN than **Group 1** **Group 3**: ↑ BV, cross‐sectional area of mid‐femur, Ct.Th, OCN, CD31; ↓ TRAP staining than **Groups 1 and 2**
Carmon 2023 [6]	6 Lewis rats. Calvarial critical size defect (8 mm diameter)	CC‐cells: Allogenic CChons (1 × 10^6^)	1: Collagen scaffold + BG (three rats) 2: Collagen scaffold + BG + cells (three rats)	3 mo: ‐ Live CT scan ‐ Micro‐CT ‐ Histology ‐ Dynamic histology	**Group 2**: ↑ BV, MAD of the defect edge region; ↓ average distance and area between the edges of the defect than **Group 1**
Zuo 2022 [57]	95 SD rats with patellar tendon, the lower 1/3 part of the patella (1 mm), and part of the tibial tubercle (1 mm) resection	CC‐cells: Allogenic BMSCs or CDSCs (3 × 10^6^)	1: DTS (25 rats) 2: BMSCs + rat DTS (35 rats) 3: CDSCs + rat DTS (35 rats)	2 mo: ‐ Histology ‐ IP ‐ Mechanical test	**Group 3**: ↑ cell density, regenerated structure, FMOD, Tnmd, Osx, ColX than **Group 2** **Group 3**: ↑ failure load, stiffness; ↓ histological appearance than **Groups 1 and 2**
*Ectopic models*					
He 2017 [20]	8 nude mice. Three subcutaneous pockets	CC‐matrix: Human decellularized CC sheets	1: CC‐matrix (eight pockets) 2: hAUC sheet (eight pockets) 3: hNSC sheet (eight pockets)	3 mo: ‐ Histology ‐ Biomechanical evaluations ‐ ELISA	**Groups 1–3**: Chondrogenic ability **Group 2**: ↑ DNA content, Elastin; ↓ COLL I than **Groups 1 and 3** **Group 2**: ↑ GAG than group 1 **Group 3**: ↑ GAG than **Groups 1 and 2** **Group 3**: ↑ Elastin than **Group 1**
Dong 2023 [12]	8 nude mice. 4 subcutaneous pockets	CC‐matrix: Human decellularized diced CC	1: Shaved CC‐matrix (eight mice); 2: CC‐matrix (diameter <0.5 mm) (eight mice) 3: CC‐matrix (diameter 0.5–1.0 mm) (eight mice) 4: CC‐matrix (diameter 1.0–1.5 mm) (eight mice)	3 mo: ‐ Volume and weight of each graft sample ‐ Volume and weight retention ‐ Histology ‐ Biomechanical analysis	**Group 4**: ↑ wet weight retention rate, regeneration potential than **Groups 1–3** **Groups 3 and 4**: ↑ elasticity modulus, chondrocyte, vascularization and collagen than **Groups 1 and 2** **Group 2**: ↑ volume retention rate, loss of chondrocytes than **Groups 3 and 4**
Zhang 2018 [51]	24 nude mice. 1 subcutaneous task	CC‐matrix: Decellularized goat CC matrix + rat BMSCs (5–10 × 10^5^)	1: CC‐matrix (6 mice) 2: SF + CC‐matrix (1:1) (6 mice) 3: SF + CC‐matrix (1:2) (six mice) 4: SF + CC‐matrix (1:4) (six mice)	18 days: ‐ Histology ‐ IHC	**Groups 1–4**: chondrogenesis, cartilage regeneration, PG, collagen **Group 3**: ↑ PG, collagen, COLL II; ↓ COLL I than **Groups 1, 2 and 4**
Isaeva 2023 [24]	Eight Wistar rats. One subcutaneous pocket	CC‐cells: Allogenic CChons	1: Hydrogel + cells (2 × 10^6^ m/L^–1^) (four rats) 2: GelMA + cells (3 × 10^6^ m/L^–1^) (four rats)	26 days: ‐ Histology ‐ IHC	**Group 1**: Scaffold in the implantation area, multinuclear macrophages; ↓ Foci of cartilage proliferation among the muscles, COLL II, COLL I **Group 2**: Island of cartilaginous tissue with a poorly developed perichondrium; ↑ COLL II, COLL I, Alcian blue

Abbreviations: BMSCs, bone marrow‐derived mesenchymal stem cells; BV, bone volume; CC, costal cartilage; CChon, costal chondrocytes; CDSCs, costal‐cartilage‐derived stem cells; CMC, carboxymethyl cellulose; COL1A1, collagen type I alpha 1 chain gene; COL2A1, collagen type II alpha 1 chain gene; COLL X, type X collagen protein; COLL I, type 1 collagen protein; COLL II, type 2 collagen protein; Ct.Th, cortical thickness; CT, computed tomography; DTS, decellularized tendon scaffold; ELISA, enzyme‐linked immunosorbent assay; FMOD, fibromodulin; F‐up, follow‐up; GAG, glycosaminoglycan; GelMA, gelatin methacrylate; hAUC, human auricular chondrocytes; hNSC, human nasoseptal chondrocytes; hWJMSC, human Wharton's jelly mesenchymal stem; IHC, immunohistochemistry; IP, immunofluorescence; MAD, mineral apposition distance; MA‐ECM, minimally manipulated adipose tissue‐extracellular matrix; MMP‐3, metalloproteinase‐3; MMP‐9, Metalloproteinase‐9; MOCART, magnetic resonance observation of cartilage repair tissue; MRI, magnetic resonance imaging; NZW, New Zeland White; OC, osteochondral; OCN, osteocalcin; PCL, poly(ε‐caprolactone); PG, proteoglycan; RT‐PCR, reverse transcription polymerase chain reaction; SD, Sprague–Dawley; SF, silk fibroin.

**Table 3:** Characteristics of the clinical studies included in the systematic review.

Ref.	Type of study: pz lesion	CC matrices/cells	Experimental groups	F‐up: Mat and met	Main outcomes
*CC‐matrix*					
Shon 2023 [36]	Retrospective comparative: 102 pz (16 M/86 F, 55.5 ± 5.3 years). Medial femoral condyle full‐thickness cartilage defect (≥200 mm^2^)	CC‐matrix: Paste of allogeneic decellularized PCHCA from cadavers mixed with normal saline and decellularized human allodermic powder	1: SD (51 pz) 2: SD + CC‐matrix (51 pz)	24 mo: ‐ Clinical scores ‐ AEs ‐ Radiography	**Groups 1 and 2**: ↑ KOOS, ROM; ↓ WOMAC **Group 2:** ↑ regenerated cartilage, ICRS‐CRA grading system, Koshino staging system than **Group 1**
Chung 2025 [8]	Retrospective cohort study: 40 pz (11 M/29 F, 56.5 ± 4.5 years). Focal cartilage defect (<1000 mm^2^)	CC‐matrix: paste‐type hyaline cartilage‐derived ECM harvested from cadaveric CC, processed via particulation and decellularization	1: HTO + MF (21 pz) 2: HTO + MF + CC‐matrix (19 pz)	12 mo: ‐ Clinical score ‐ Radiography	**Groups 1 and 2**: ↑ IKDC score, KOOS, ADL, QOL, posterior tibial slope; ↓ HKA angle, VAS **Group 2**: ↑ repaired cartilage quality, ICRS‐CRA grading system and Koshino staging system than **Group 1**
Zhang 2022 [49]	Prospective study: 20 pz (12 M/8 F, 31 ± 7.2 years). Femoral head OC lesion (>300 mm^2^)	CC‐matrix: Autologous CC without perichondrium fixed using 2 absorbable screws	1: CC‐matrix (20 pz)	36 mo: ‐ Clinical scores ‐ MOCART	**Group 1**: ↑ HHS, EQ VAS, UCLA; ↓ MOCART; biochemical constituents of the implanted graft = hyaline cartilage No major local or systemic complication
Wei 2023 [43]	Prospective study: 5 pz (5 M, 36.6 ± 11.1 years). OLT with subchondral cysts	CC‐matrix: Autologous costal osteochondral graft	1: CC‐matrix (5 pz)	12 mo: ‐ Clinical scores	**Group 1**: No pain, sports level reached the expected level, no major AE ↓ NRS; ↑ Tegner score, AOFAS score, FAAM score Complete defect filling, good integration, similar signal to native cartilage, hyaline chondroid tissue
Chung 2023 [9]	RCT: 88 pz (29 M/59 F, 54 ± 5.9 years). Focal cartilage defect (<1000 mm^2^)	CC‐matrix: paste‐type hyaline cartilage‐derived ECM harvested from cadaveric CC, processed via particulation and decellularization	1: MF (44 pz) 2: MF + CC‐matrix (44 pz)	12 mo: ‐ MRI ‐ Clinical scores ‐ Safety evaluation	**Group 2**: ↑ MOCART, KOOS pain, KOOS symptoms, ADL and Sport; ↓ VAS pain than **Group 1**
*CC‐cells*					
Yoon 2024 [45]	RCT: 25 pz (16 M/9 F, 43.8 ± 8.7 years). Cartilage defect (200–1000 mm^2^) on medial or lateral femoral condyle or trochlea	CC‐cells: Autologous CChons pellet (1 × 10^5^ cells/well)	1: MF (9 pz) 2: Cell pellets + fibrin glue (16 pz)	60 mo: ‐ Clinical scores ‐ MOCART ‐ Failure ‐ Safety	**Group 2**: ↑ Lysholm, KOOS, MOCART than Group 1) **Group 1**: HTO (1pz) **Group 2**: ACL rupture during f‐up (1 pz) No AE related to the CC harvesting procedure
Yoon 2021 [47]	RCT: 30 pz (17 M/13 F, 44.4 ± 11.9 years). Chondral defect (200–1000 mm^2^)	CC‐cells: Autologous CChons pellet (1 × 10^5^ cells/well)	1: MF (10 pz) 2: Cell pellets + fibrin glue (20 pz)	12 mo: ‐ MOCART ‐ Clinical scores	**Groups 1 and 2**: ↑ MOCART, Lysholm, IKDC, KOOS; ↓ VAS pain **Group 2**: ↑ MOCART, defect repair degree, filling of the defect, integration to border zone, surface intact, Lysholm, KOOS sport, KOOS QOL than **Group 1**
Yoon 2020 [46]	Case series: 7 pz (4 M/3 F, 40 ± 8.7 years). Focal chondral defect (200–1000 mm^2^)	CC‐cells: Autologous CChons pellet (1 × 10^5^ cells/well)	1: Cell pellets + fibrin glue (7 pz)	60 mo: ‐ Clinical scores ‐ MRI	**Group 1**: ↑ IKDC subjective, Lysholm, Tegner activity, MOCART

Abbreviations: ACAN, Aggrecan gene; ACI, autologous chondrocyte implantation; ADL, activities of daily living; AEs, Adverse events; AOFAS, American Orthopedic Foot & Ankle Society score; AUC, auricular cartilage; BG, bone graft; BMSCs, bone marrow‐derived mesenchymal stem cells; BV, bone volume; CC, costal cartilage; CChon, costal chondrocytes; CDSCs, costal‐cartilage‐derived stem cells; COL1A1, collagen type I alpha 1 chain gene; COL2A1, collagen type II alpha 1 chain gene; COLL X, type X collagen protein; COLL I, type 1 collagen protein; COLL II, type 2 collagen protein; Ct.Th, cortical thickness; CT, computed tomography; DTS, decellularized tendon scaffold; ECM, extracellular matrix; EQ VAS, EuroQol visual analogue scale; FAAM, Foot and Ankle Ability Measure score; FMOD, fibromodulin; GAG, glycosaminoglycan; GelMA, gelatin methacrylate; HA‐CMC, hyaluronic acid–carboxymethyl cellulose; hAUC, human auricular chondrocytes; HHS, Harris Hip Score; HKA, hip–knee–ankle; hNSC, human nasoseptal chondrocytes; HTO, high tibial osteotomy; hWJMSC, human Wharton's jelly mesenchymal stem; ICRS, International Cartilage Repair Society; ICRS‐CRA, International Cartilage Repair Society‐Cartilage Repair Assessment; IHC, immunohistochemistry; IKDC, International Knee Documentation Committee; IP, immunofluorescence; KOOS, Knee injury and Osteoarthritis Outcome score; MAD, mineral apposition distance; MA‐ECM, minimally manipulated adipose tissue‐extracellular matrix; MF, microfracture; MFC, medical femoral condyle; MMP‐3, metalloproteinase‐3; MMP‐9, Metalloproteinase‐9; MOCART, magnetic resonance observation of cartilage repair tissue; MOCART, magnetic resonance observation of cartilage repair tissue; MRI, magnetic resonance imaging; NRS, numeric rating score; NZW, New Zeland White; OC, osteochondral; OCN, osteocalcin; OLT, osteochondral lesions of the talus; OSX, osterix; PCHCA, particuled costal hyaline cartilage allograft; PCL, poly(ε‐caprolactone); PG, proteoglycan; QOL, quality of life; RCT, randomized controlled trial; ROM, range of motion; RUNX2, runt‐related transcription factor 2 gene; SD, Sprague–Dawley; SD, subcondral drilling; SDSCs, synovium‐derived stromal cells; SF, silk fibroin; SOX9, SRY‐box transcription factor 9 gene; TGF‐β3, Transforming growth factor beta‐3; Tnmd, tenomodulin; UCLA, University of California Los Angeles scale; VAS, visual analogue scale; WOMAC, Western Ontario and McMaster University.

### Risk of bias assessment

Two authors (F. V. and L. C.) independently performed the assessments, and discrepancies were resolved by consensus or consultation with a third reviewer (M. G.).

The methodological quality of the included in vivo studies was evaluated using the Systematic Review Centre for Laboratory Animal Experimentation (SYRCLE)'s Risk of Bias (RoB) tool, specifically developed for animal intervention studies [22]. This tool is based on the Cochrane Collaboration's RoB tool but adapted to account for characteristics specific to animal research. It assesses potential bias across six domains: (1) random sequence generation, (2) allocation concealment, (3) blinding (personnel/outcome), (4) incomplete outcome data, (5) selective reporting and (6) other sources of bias. Each domain was judged as low, unclear or high risk of bias according to the criteria proposed by SYRCLE. When information was insufficient to make a clear judgement, the risk of bias was classified as unclear. The results of the assessment were summarized in graphical form to illustrate the overall quality of the in vivo evidence.

As regards clinical studies, the methodological quality was assessed using the Cochrane risk‐of‐bias tools, according to study design. For randomized controlled trials (RCTs), the Cochrane Risk of Bias 2.0 (RoB 2) tool was applied, which evaluates five domains: (1) bias arising from the randomization process, (2) bias due to deviations from intended interventions, (3) bias due to missing outcome data, (4) bias in measurement of the outcome and (5) bias in selection of the reported result. Each domain was rated as low, moderate or high risk of bias, following the Cochrane Handbook recommendations [37].

For non‐randomized and observational studies, the Risk Of Bias in non‐randomized studies of interventions (ROBINS‐I) tool was used [38]. This tool assesses bias across seven domains: (1) confounding, (2) selection of participants into the study, (3) classification of interventions, (4) deviations from intended interventions, (5) missing data, (6) measurement of outcomes and (7) selection of the reported result. Judgements were made as low, moderate or high risk of bias. The results of the assessment were summarized in graphical form to illustrate the overall quality of the clinical evidence.

## RESULTS

This systematic review has been registered on PROSPERO (ID number CRD420261294029).

### Included and excluded studies

**Figure 1:**
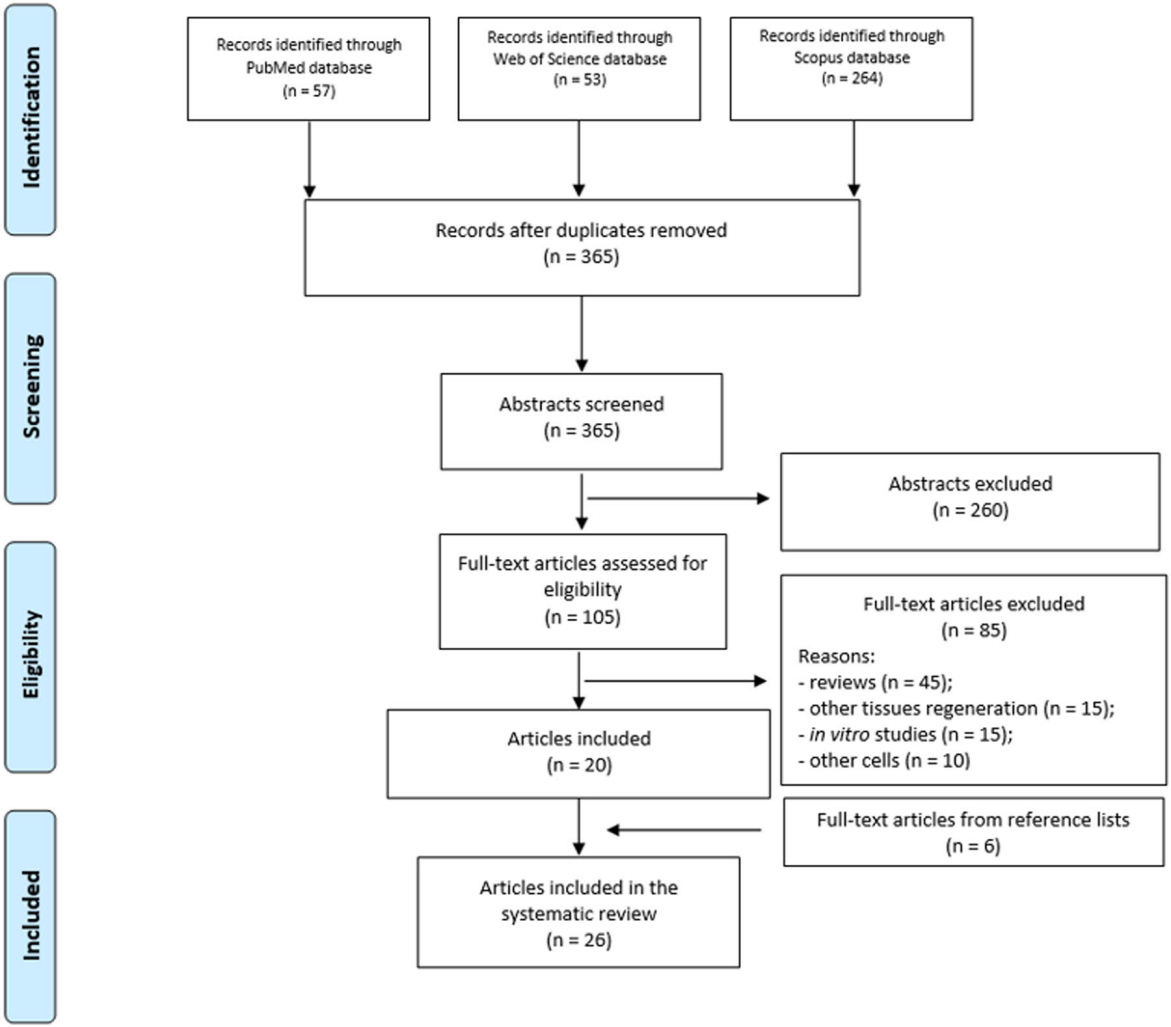
The PRISMA flow diagram for the systematic review detailing the database searches, the number of abstracts screened and the full texts retrieved. PRISMA, Preferred Reporting Items for Systematic Reviews and Meta‐Analyses.

Figure 1 illustrates the systematic review details of database searches. Of these, 18 were in vivo investigations [4, 5, 6, 7, 12, 13, 18, 20, 21, 24, 26, 27, 33, 51, 53, 54, 55, 57] and 8 were clinical ones [8, 9, 36, 43, 45, 46, 47, 49].

### In vivo studies

In vivo evidence primarily addressed cartilage defect regeneration (nine studies) [5, 7, 12, 18, 20, 24, 33, 51, 53], followed by osteochondral (five studies) [13, 26, 27, 54, 55], bone (three studies) [4, 6, 21] and enthesis (one study) [57] repair models (Table 2).

The included in vivo studies were characterized by substantial heterogeneity in terms of (i) animal species (dogs [33], rabbits [7, 13, 18, 26, 53], rats [5, 6, 21, 24, 27, 54, 55, 57] and mice [4, 12, 20, 51]), (ii) defect type (full‐thickness [5, 33, 53, 57], focal [7, 18], OC [13, 26, 27, 54, 55], segmental [4, 21], critical size [6], subcutaneous implantations [12, 20, 24, 51]), (iii) implantation site (medial femoral condyle [33], rib [7, 18], grove centre [5, 13, 26, 27, 54, 55], mandibular angle [21], femur [4], calvaria [6], enthesis [57]), (iv) and type of CC‐based intervention (CC‐matrices [7, 12, 13, 18, 20, 21, 26, 33, 51, 55] or CC‐cells [4, 5, 6, 24, 27, 53, 54, 57]).

To enable a more structured interpretation, studies were stratified into orthotopic models (anatomically relevant defect sites) and ectopic models (subcutaneous implantation) and further categorized according to matrix‐based versus cell‐based approaches.

#### Orthotopic models

Most in vivo studies (14/18) were conducted in orthotopic settings, including cartilage [5, 7, 18, 33, 53], osteochondral [13, 26, 27, 54, 55], bone [4, 6, 21] and enthesis [57] defects.

Across cartilage and osteochondral defect models, matrix‐based strategies (e.g., decellularized [7, 13, 18, 26] or particulated [33, 55] CC scaffolds) consistently supported defect filling, hyaline‐like tissue formation, improved biomechanical properties and reduced metalloproteinases production compared with untreated controls [7, 13, 18, 26, 33, 55]. These effects were further enhanced when CC matrices were combined with adjunctive procedures, such as microfracture (MF) [26], or the addition of ascorbate (Asc‐2P) [55], suggesting a synergistic regenerative response rather than a purely scaffold‐driven effect. In addition, similar results were observed compared to auricular cartilage (AUC).

In bone defect models, CC‐derived matrix, composed of autologous particulate CC, improved bone defect repair and bone volume (BV) parameter more than no treatment and carbonate apatite graft, showing similar results to autologous bone graft (ABG) [21].

In parallel, in cartilage or osteochondral defects, cell‐based approaches, using autologous or allogenic CChons [5, 27, 53, 54], demonstrated robust regenerative potential, particularly when delivered within hydrogels [5, 53] or pellets [27, 54]. These strategies were associated with increased type II collagen deposition, improved matrix organization and superior histological and biomechanical scores compared with cell‐free constructs [5, 53]. Notably, co‐culture systems with Wharton's jelly MSCs (hWJMSC) [54] or synovium‐derived stromal cells (SDSCs) [27] appeared to further enhance tissue quality and reduce hypertrophic features.

Moreover, cell‐based constructs, characterized by allogenic CChons [6] or CDSCs [4] seeded onto collagen scaffolds, promoted new bone formation and structural integration, supporting the concept that CC may contribute to regeneration through endochondral‐like mechanisms in bone defects.

Similarly, the single enthesis study demonstrated that allogenic CDSCs onto decellularized tendon scaffold (DTS) improved fibrocartilaginous organization and mechanical strength compared with bone marrow mesenchymal stem cells (BMSCs) or scaffold alone [57].

#### Ectopic models

A subset of studies (4/18) [12, 20, 24, 51] employed ectopic subcutaneous implantation models to evaluate the intrinsic chondrogenic potential of CC‐derived materials.

Human or goat decellularized CC matrices increased chondrogenic ability, especially when they were combined with silk fibroin (SF) scaffold [51], or when CC matrices had a diameter over 0.5 mm, but to a lesser extent than scaffolds characterized by AUC or nasoseptal chondrocytes (NSC).

Finally, allogenic CChons entrapped in gelatine methacrylate (GelMA) scaffold, improved the formation of island of cartilaginous tissue with high COLL II and Alcian blue [24].

##### Clinical studies

The clinical evidence included eight studies, with variability in: (i) study design (cohort studies [36, 46, 47, 49], RCTs [8, 9, 45] and case series [43]), (ii) treatment strategies (CC‐matrices [8, 9, 36, 43, 49] or CC‐cells [45, 46, 47]) and (iii) concomitant surgical procedures (subchondral drilling [SD] [36], MF [8, 9] or high tibial osteotomy [HTO] [8]).

Six of them focused on cartilage repair [8, 9, 36, 45, 46, 47] and two on osteochondral reconstruction [43, 49] (Table 3).

#### Matrix‐based approaches

As regards cartilage defects, three studies investigated decellularized or particulate allogenic CC‐derived matrices, typically used as adjuncts to standard surgical procedures such as MF [8, 9], SD [36] or HTO [8].

Across these studies, consistent improvements were observed in patient‐reported outcomes, imaging‐based repair quality (MOCART scores) and macroscopic cartilage appearance compared with surgery alone, up to 24 months.

Two additional studies explored CC‐based grafts for osteochondral defect reconstruction [43, 49]. These demonstrated consistent defect filling, hyaline‐like tissue formation and functional recovery, with no major complications reported, also at 36 months, further supporting the versatility of CC in different musculoskeletal contexts.

#### Cell‐based approaches

As regards cartilage defects, three studies evaluated autologous CChon‐based therapies, primarily delivered as pellet‐type constructs [45, 46, 47].

These studies reported durable improvements in functional outcomes, defect filling and integration [45, 46, 47], often superior to MF alone [45, 47]. Importantly, cell‐based approaches appeared to provide more sustained repair over time (60 months), suggesting a potential advantage in long‐term tissue quality.

### Risk of bias

**Figure 2:**
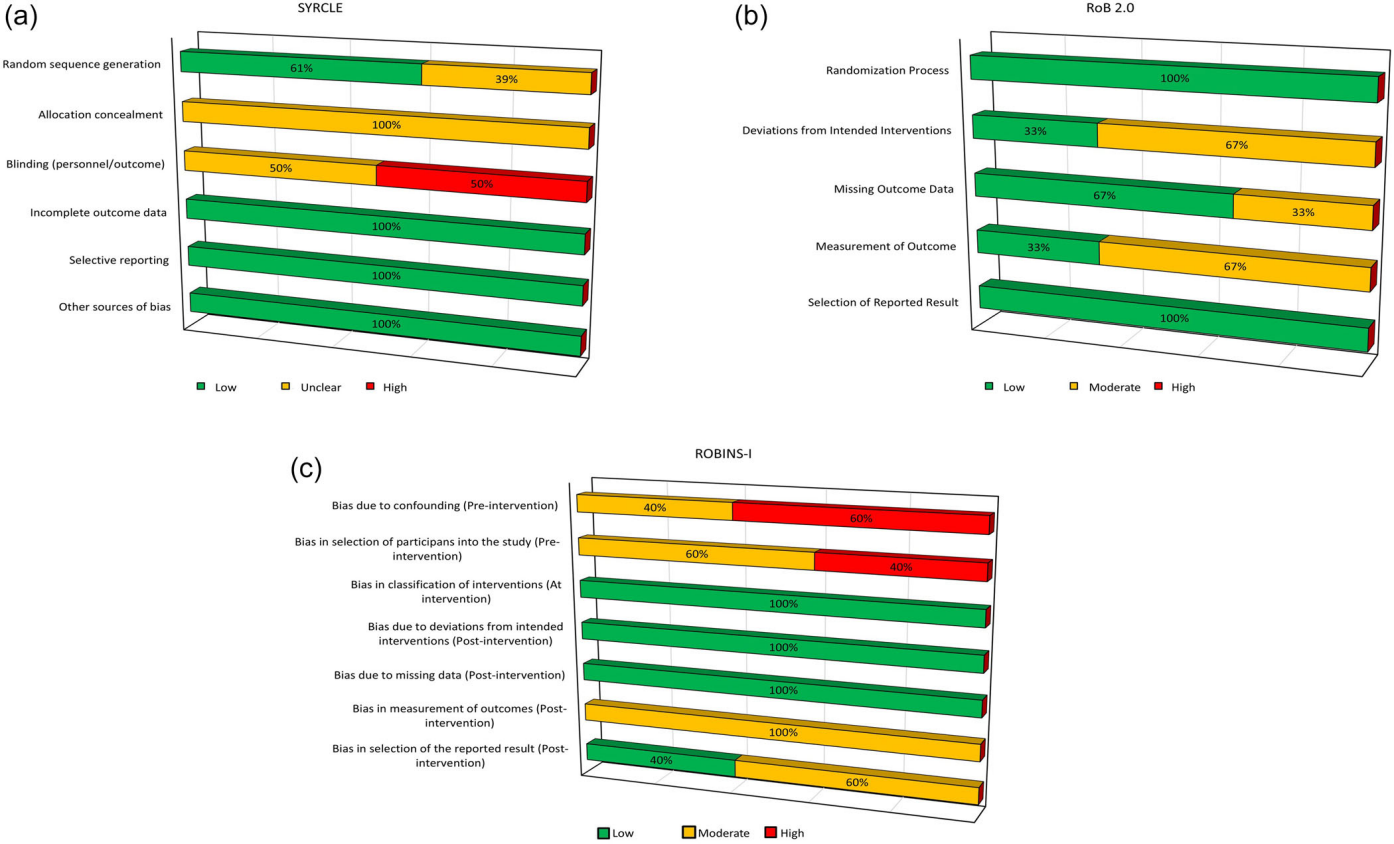
Risks of bias for in vivo and clinical studies. (a) SYRCLE risk of bias for in vivo studies: low (green), unclear (yellow) and high (red) risk of bias for each domain; (b) risk of bias summary for RCT (RoB 2.0): low (green), moderate (yellow) and high (red) risk of bias for each domain; (c) ROBINS‐I risk of bias for non‐randomized studies: low (green), moderate (yellow) or high (red) risk of bias for each domain. RCT, randomized controlled trial; ROBINS‐I, Risk Of Bias In Non‐randomized Studies—of Interventions; SYRCLE, Systematic Review Centre for Laboratory Animal Experimentation.

The risk of bias assessment using the SYRCLE tool revealed an overall moderate methodological quality across the included in vivo studies. As shown in Figure 2, the domains of random sequence generation and allocation concealment were most often rated as ‘unclear risk’: approximately 39% [6, 7, 12, 13, 18, 24, 57] and 100%, respectively. This reflects the incomplete reporting of randomization methods and group allocation procedures. The blinding of personnel and outcome assessors represented the domain with the highest risk of bias, as 50% of studies [4, 6, 7, 12, 18, 24, 26, 55, 57] did not specify whether blinding was implemented during experimental procedures or histological evaluation. In contrast, incomplete outcome data, selective reporting and other sources of bias were predominantly judged as low risk, indicating appropriate handling of data, comprehensive reporting of outcomes and adherence to ethical and experimental standards. Overall, these findings suggest that while the included animal studies were generally well conducted, they often lacked sufficient methodological transparency in randomization and blinding procedures (Figure 2a).

As regards clinical studies, the assessment of RCTs (RoB 2.0) showed an overall low to moderate risk of bias across the evaluated domains. Specifically, all studies presented a low risk in both the randomization process and the selection of reported results. However, some concerns were identified regarding deviations from the intended interventions, missing outcome data and outcome measurement. These were 67% [43, 47], 33% [47] and 67% [43, 47], respectively. No trials were judged at high risk for any domain, indicating overall good methodological quality among randomized studies (Figure 2b).

For non‐randomized and observational studies assessed with the ROBINS‐I tool, the overall methodological quality was moderate. The highest concerns were related to confounding, 60% high [8, 46, 49] and 40% moderate risks [9, 36], and participant selection, 60% moderate [9, 36, 43] and 40% high [8, 46]. By contrast, all studies were rated as having low bias relating to intervention classification, deviations from intended interventions and missing data. The measurement of outcomes and selective reporting were rated as moderate 100% and 60% [8, 43, 46], respectively). Collectively, these findings suggest that while randomized studies demonstrated acceptable internal validity, non‐randomized evidence was affected by residual confounding and selection biases (Figure 2c).

## DISCUSSION

The present systematic review provides a comprehensive overview of the current in vivo and clinical evidence, supporting the use of CC, in both cellular and matrix‐based forms, for musculoskeletal tissue regeneration. By focusing on studies published over the last decade and excluding in vitro‐only investigations, this review highlights the translational potential of CC‐derived strategies across cartilage, osteochondral, bone and enthesis repair. The findings suggest that CC may represent a versatile and biologically effective resource for promoting tissue regeneration, with generally favourable structural, biochemical and functional outcomes reported across.

This versatility is underpinned by several intrinsic biological and translational advantages of CC compared with other cartilage sources, such as articular, auricular or nasal cartilage (NSC), that support its greater versatility in musculoskeletal regeneration. As permanent hyaline cartilage, it closely resembles articular cartilage in composition while providing higher cell yield, phenotypic stability and broader availability with lower donor‐site morbidity [23]. Unlike elastic cartilages, CC lacks elastin‐rich networks that may compromise mechanical performance in load‐bearing environments [20]. Importantly, its intrinsic biological plasticity and responsiveness to both chondrogenic and osteogenic cues, likely related to its developmental similarity to growth plate cartilage and tendency toward hypertrophy and ossification, make it particularly suitable for osteochondral, bone and interface tissue repair [21, 55]. From a translational standpoint, CC is also more amenable to standardization and banking, especially in its decellularized form, further enhancing its clinical relevance [9, 26].

One of the most striking observations emerging from this review is the breadth of musculoskeletal applications for which CC‐based approaches have shown efficacy. While most included studies focused on articular cartilage repair [5, 7, 8, 9, 12, 18, 20, 24, 33, 36, 45, 46, 47, 51, 53], CC‐cells or matrices were also successfully applied to osteochondral defects [13, 26, 27, 43, 49, 54, 55], critical‐size bone defects [4, 6, 21] and complex interface tissues such as the enthesis [57]. This wide applicability likely reflects the intrinsic biological properties of CC, including its hyaline cartilage composition, high cellularity in younger donors and developmental proximity to endochondral ossification pathways.

In cartilage orthotopic and ectopic repair models, both CC‐derived matrices and CChons consistently supported the formation of hyaline‐like tissue [5, 7, 8, 33, 36, 47, 53], characterized by high COLL II [7, 24, 33, 51, 53] and GAG content [20], low COLL I expression [5, 7, 20, 24, 33] and improved radiographic [33], macroscopic [5, 33, 53] and biomechanical properties [5, 12]. These outcomes were observed across multiple animal species and defect models, suggesting consistent indications of chondrogenic potential across different models, although variability in study design should be considered.

In osteochondral models, CC‐derived matrices facilitated not only chondral resurfacing but also integration with subchondral bone [13, 26, 55], while CChons promoted coordinated regeneration when combined with supportive cell populations [27, 54]. Similarly, in bone defect models, CC grafts and CC‐derived cells supported new bone formation, likely through endochondral mechanisms that mirror physiological bone development [4, 6, 21]. These findings collectively support the concept that CC is not merely an alternative cartilage source but a potentially versatile regenerative substrate that may be applicable across different musculoskeletal settings, although further validation is needed.

The in vivo studies included in this review confirmed that CC‐based approaches are capable of inducing cartilage‐like tissue formation or bone integration in a controlled environment. However, variability in scaffold composition, cell delivery strategies and defect characteristics contributed to differences in the magnitude of regenerative outcomes.

Compared with orthotopic models, ectopic systems provided more controlled biological insights but limited information regarding functional integration and biomechanical performance.

From a translational perspective, ectopic models offer lower evidentiary weight, as they do not replicate the complex mechanical and biological environment of native musculoskeletal tissues. When considering study design and model relevance, orthotopic studies provide more clinically meaningful evidence, whereas ectopic models mainly support mechanistic understanding.

Across both settings, cell‐based approaches generally yielded stronger regenerative responses, albeit with greater complexity, while matrix‐based strategies offered more consistent and translatable outcomes, particularly when used in combination with surgical techniques.

The clinical studies included in this review provide encouraging evidence supporting the safety and efficacy of CC‐based therapies in human patients. Across these studies, recurring patterns include significant functional improvement, good defect filling and favourable safety outcomes. However, key discrepancies arise from differences in concomitant procedures, treatment protocols and outcome measures. When stratified by study design, RCTs [9, 45, 47] provided the most robust evidence, generally supporting the efficacy of CC‐based therapies, particularly for cell‐based approaches [45, 47]. In contrast, non‐randomized and observational studies were more susceptible to confounding and selection bias, as also reflected in the risk of bias assessment [8, 36, 43, 46, 49].

Clinical evidence highlights the need for standardized protocols and high‐quality trials specifically designed to isolate the effects of CC‐based therapies.

The interpretation of matrix‐based approaches is limited by the combined use of surgical techniques, which likely contributed to the observed effects. Despite this limitation, matrix‐based strategies demonstrated reproducible clinical benefits and a favourable safety profile, supporting their potential as readily translatable therapeutic options. Compared with matrix‐based strategies, cell‐based approaches involve greater procedural and regulatory complexity.

Across cartilage and osteochondral defects, CC‐derived matrices and cells were associated with significant improvements in patient‐reported outcome measures, imaging‐based repair scores and functional recovery [8, 9, 36, 45, 46], with follow‐up periods extending up to 5 years in some trials [45, 46]. Notably, autologous CChon implantation demonstrated durable results without major donor‐site morbidity, addressing a key concern traditionally associated with rib cartilage harvesting [43, 45, 46, 47, 49]. Importantly, several studies demonstrated repair tissue quality comparable to or superior to that achieved with conventional techniques such as MF, which is known to favour fibrocartilaginous repair [8, 9, 45, 47].

The favourable safety profile observed across studies is particularly relevant for clinical translation. Neither allogeneic CC matrices nor autologous CC‐derived cell therapies were associated with severe local or systemic complications, immune reactions or graft failures [43, 45, 49]. These findings align with the established clinical use of CC in craniofacial and reconstructive surgery and support its broader application in orthopaedic and sports medicine contexts [15, 30].

A key theme emerging from the reviewed literature is the comparison between cell‐based and matrix‐based CC‐derived therapies. Cell‐based approaches, employing either CChons [4, 5, 24, 27, 45, 46, 47, 53, 54] or CDSCs [4, 57], demonstrated encouraging regenerative outcomes, particularly when cells were delivered in pellets [27, 45, 46, 47, 54], hydrogels [24, 53] or composite scaffolds [4, 5, 6, 24, 57]. These strategies often resulted in enhanced matrix deposition, improved histological scores and superior functional outcomes compared with cell‐free controls. Moreover, co‐culture systems involving CC‐derived cells and other progenitor populations, such as BMSCs [5], SDSCs [27] or WJMSCs [54], appeared particularly effective in mitigating hypertrophy and improving tissue organization.

However, despite their biological efficacy, cell‐based strategies are associated with significant translational and regulatory challenges. The isolation, expansion and manipulation of CC‐derived cells typically qualify as substantial manipulation, placing these therapies within the regulatory framework of ATMPs in many countries. This classification increases manufacturing complexity, costs and regulatory burden, potentially limiting widespread clinical adoption [28, 34, 44].

In contrast, matrix‐based approaches using decellularized or particulated CC scaffolds offer a more readily translatable option. These materials retain the native extracellular matrix architecture and bioactive cues necessary to support endogenous cell recruitment and differentiation, often without the need for exogenous cells or growth factors [26]. Nevertheless, it is worth noting that when CC is used as allogeneic tissue or as scaffold, accredited tissue banks, along with rigorous protocols for sterile manipulation, microbial testing, decellularization, sterilization or cryopreservation, are essential to ensure safety, reproducibility and regulatory compliance. Furthermore, variability in donor age, tissue quality and decellularization methods represents critical parameters that must be carefully considered, as they may introduce bias and affect efficacy, standardization and reproducibility.

Most of such clinical evidence originates from Asia, particularly South Korea, where tissue banking and regulations differ from those in the West. In Europe and the United States, CC is mainly used as a structural graft for procedures like rhinoplasty [15] and auricular reconstruction [39]. Regulatory frameworks shape the use of processed or engineered cartilage: in the United States, minimally manipulated grafts follow the FDA's 361 HCT/P pathway (https://www.ecfr.gov/current/title-21/chapter-I/subchapter-L/part-1271), while more extensively processed products require biologics approval; in Europe, strict EU (https://eur-lex.europa.eu/eli/dir/2004/23/oj/eng) and EDQM standards (https://www.edqm.eu/en/) apply, and substantially manipulated or non‐homologous tissues are regulated as ATMPs. In contrast, in South Korea, the Act on Advanced Regenerative Medicine and Advanced Biological Products (ARMAB) (https://elaw.klri.re.kr/eng_mobile/viewer.do?hseq=56486&type=part&key=37) actively supports both autologous and allogeneic CC in regenerative applications, providing streamlined pathways for tissue‐engineered therapies.

The interpretation of the findings of this systematic review should be carefully considered in light of the methodological quality of the included studies. Although overall results consistently suggest a beneficial role of CC in musculoskeletal regeneration, the confidence in these findings is moderated by the risk of bias assessment. In particular, in vivo studies frequently presented unclear risk of bias in key domains, such as allocation concealment and blinding, which may have contributed to an overestimation of the treatment effects.

Similarly, non‐randomized clinical studies were substantially affected by confounding and selection bias, limiting the internal validity and generalizability of their outcomes. Therefore, while the available evidence is encouraging, greater weight should be attributed to RCTs, which demonstrated comparatively lower risk of bias, whereas findings from observational and preclinical studies should be interpreted with caution. These considerations underline the need for more rigorously designed studies to strengthen the reliability and clinical applicability of the current evidence base.

In addition, a relevant limitation emerging from the analysis of clinical studies is that CC‐based interventions were sometimes applied in combination with established surgical procedures, such as MF [8, 9], SD [36] or HTO [8], rather than as standalone treatments. While this combined approach reflects current clinical practice and enhances the translational applicability of these strategies, it introduces a significant source of confounding. Specifically, the concomitant procedures themselves are known to promote cartilage repair and functional improvement, thereby making it challenging to isolate the independent contribution of CC‐derived matrices or cells. Consequently, the beneficial outcomes reported across these studies may partly result from synergistic effects between the surgical techniques and the CC‐based therapies, rather than from the latter alone. This limitation should be carefully considered when interpreting the clinical efficacy of CC‐based approaches, and it underscores the need for future RCTs specifically designed to disentangle their individual effects through appropriate control groups and standardized treatment protocols.

Finally, it is important to acknowledge that most clinical evidence remains limited to relatively small cohorts (from a minimum of 7 to a maximum of 102 patients) and short‐ to mid‐term follow‐up periods (from a minimum of 12 to a maximum of 60 months). While early and intermediate outcomes are promising, longer‐term data are needed to confirm the durability of regenerated tissue, particularly under high mechanical loading conditions and in younger, active patient populations.

Future research should aim to clarify several outstanding questions regarding CC‐based musculoskeletal regeneration. Comparative studies directly evaluating CC against other cartilage sources, such as articular or NSC, would help define its relative advantages. Additionally, further exploration of donor age, tissue processing methods and scaffold architecture may optimize regenerative outcomes. From a translational standpoint, well‐designed multicenter RCTs with long‐term follow‐up are essential to establish the definitive clinical role of CC‐derived therapies. Furthermore, a search of clinical trial registries, including ClinicalTrials.gov and other international trial databases, revealed no ongoing studies specifically designed to address these limitations or to provide high‐level clinical evidence for CC‐based musculoskeletal regenerative approaches.

## CONCLUSIONS

In conclusion, the evidence synthesized in this systematic review suggests that CC may represent a promising and versatile resource for musculoskeletal tissue regeneration. Both cell‐based and matrix‐based approaches have shown encouraging results in vivo and early clinical settings. However, these findings should be interpreted with caution due to the heterogeneous methodological quality of the included studies, and further high‐quality RCTs are required to confirm their clinical effectiveness.

## AUTHOR CONTRIBUTIONS

*Conceptualization*: Francesca Veronesi and Marco Govoni. *Methodology*: Francesca Veronesi and Luca Cavazza. *Formal analysis and investigation*: Leonardo Vivarelli, Gianluca Giavaresi and Marta Pluchino. *Writing—original draft preparation*: Francesca Veronesi, Luca Cavazza and Marco Govoni. *Writing—review and editing*: Francesca Veronesi and Marco Govoni. *Funding acquisition*: Dante Dallari. *Supervision*: Marco Govoni.

## CONFLICT OF INTEREST STATEMENT

The authors declare no conflicts of interest.

## ETHICS STATEMENT

The authors have nothing to report.

## Data Availability

The data that support the findings of this study are available from the corresponding author upon reasonable request.
